# Design, Modeling and Synthesis of 1,2,3-Triazole-Linked Nucleoside-Amino Acid Conjugates as Potential Antibacterial Agents

**DOI:** 10.3390/molecules22101682

**Published:** 2017-10-10

**Authors:** Sarah N. Malkowski, Carolyn F. Dishuck, Gene G. Lamanilao, Carter P. Embry, Christopher S. Grubb, Mauricio Cafiero, Larryn W. Peterson

**Affiliations:** Department of Chemistry, Rhodes College, 2000 North Parkway, Memphis, TN 38112, USA; snmalkowski@gmail.com (S.N.M.); cdishuck@gmail.com (C.F.D.); gene.lamanilao@gmail.com (G.G.L.); embcp-19@rhodes.edu (C.P.E.); csg2140@cumc.columbia.edu (C.S.G.); cafierom@rhodes.edu (M.C.)

**Keywords:** click chemistry, CuAAC, 1,2,3-triazole, nucleoside, LpxC, antibacterial

## Abstract

Copper-catalyzed azide-alkyne cycloadditions (CuAAC or click chemistry) are convenient methods to easily couple various pharmacophores or bioactive molecules. A new series of 1,2,3-triazole-linked nucleoside-amino acid conjugates have been designed and synthesized in 57–76% yields using CuAAC. The azido group was introduced on the 5′-position of uridine or the acyclic analogue using the tosyl-azide exchange method and alkylated serine or proparylglycine was the alkyne. Modeling studies of the conjugates in the active site of LpxC indicate they have promise as antibacterial agents.

## 1. Introduction

Copper-catalyzed azide-alkyne cycloaddition (CuAAC) reactions, also commonly known as click chemistry, are widely used in a variety of interdisciplinary fields such as organic synthesis, combinational chemistry, drug development, materials science, and bioorthogonal chemistry [1]. They require an azide and an alkyne to form a 1,2,3-triazole ring in the presence of Cu(I) ions. The use of CuAAC in bioorthogonal chemistry offers a multitude of advantages. The necessary alkyne and azide moieties share properties that are ideal under the conditions of bioorthogonal chemistry, such as their small size, stability, and absence within biological systems, preventing the disruption of native chemical reactions and structures within the studied system. The CuAAC reaction has fast kinetics and high yield [1]. The stability of the signature 1,2,3-triazole ring also protects the bioactive conjugates from metabolic degradation and allows for tolerance of a diverse range of reaction conditions, chemical environments, and reagents [1,2]. 

In addition to facilitating bioorthogonal chemical reactions, a broad assortment of triazole derivatives readily synthesized via CuAAC have shown promising results within the field of medicinal chemistry, with certain triazoles displaying antifungal activity against several *Candida* species [3]. Additionally, CuAAC has made it possible for researchers to prepare novel phospholipid-protein conjugates with high binding affinity for autoimmune antibodies [4]. Recent studies have described the use of 1,2,3-triazolyl nucleosides and nucleoside analogues as chitin synthase inhibitors [5], phosphoglycosyltransferase inhibitors [6] and anticancer [7,8,9], antiviral [10,11] and antimicrobial agents [12,13,14]. These developments, as well as the results described in this paper, highlight the practicality and versatility of the CuAAC reactions.

Although there have been many great technological advances in the health industry, the treatment of microbial diseases, including bacterial, viral, and parasitic infections, remains a challenge [15]. Among the culprits of such infections are Gram-negative bacteria, such as *E. coli* and *P. aeruginosa* [16,17,18]. To further complicate problems, multidrug-resistant strains of Gram-negative bacteria present potential serious health issues [18]. The bacterial resistance and difficulty in treating Gram-negative bacterial infections have prompted researchers to develop novel and effective antibacterial therapies [17].

The outer membrane of Gram-negative bacteria is composed of lipopolysaccharide (LPS). An integral component of LPS is lipid A, which is a glucosamine-based phospholipid. Lipid A anchors LPS to the outer membrane and is essential for the growth and viability of the bacterium [17]. There are nine enzymes involved in the biosynthetic pathway of Lipid A [18]. Among these enzymes is UDP-3-*O*-(*R*-3-hydroxymyristoyl)-*N*-acetylglucosamine deacetylase (LpxC). LpxC is responsible for catalyzing the first committed step in the synthesis of lipid A [18], and therefore, it is an attractive target for inhibiting the biosynthesis of lipid A, resulting in a compromised outer membrane.

**Figure 1:**
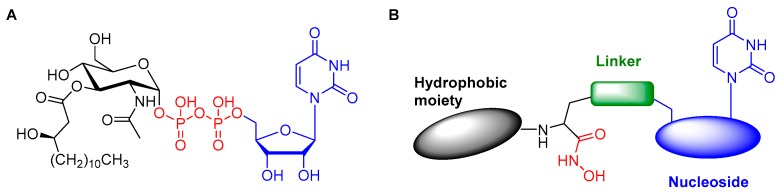
(**A**) The structure of the natural substrate of LpxC; (**B**) General structure of the triazole-linked conjugates.

Information from previous inhibitors and the structure of the natural substrate influenced rational design of the conjugates’ structures. The natural substrate features three regions: a sugar with a hydrophobic tail (black), a zinc-binding motif (red), and a uracil-based nucleoside (blue) (Figure 1A). The initial design of the conjugates featured a hydroxamic acid to bind to the zinc ion and a uracil-based nucleoside (Figure 1B). The hydrophobic moiety was initially omitted for synthetic ease. The analogues are synthesized in two building blocks, the nucleoside block and the hydrophobic moiety with the hydroxamic acid, and then clicked together to form the triazole linkage (Figure 1B).

**Figure 2:**
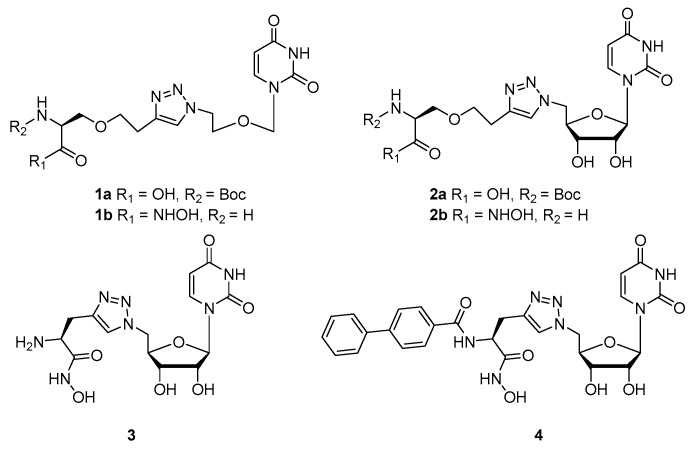
The structures of the nucleoside-amino acid conjugates.

Herein, we report the design, computational analysis and synthesis of four novel nucleoside-amino acid conjugates **1a**, **2a**, **3** and **4** (Figure 2) conveniently coupled via CuAAC, positioning a 1,2,3-triazole group in the center of the molecule, linking the nucleoside and amino acid. We chose to use amino acids because their properties can be easily modified and the carboxylic acid can be conveniently converted to a hydroxamic acid.

Not only does the use of the click reaction provide the above-mentioned advantages, these compounds or their derivatives could be further functionalized to include additional amino acids or groups off the amino group of serine (Ser) or propargylglycine that would further modulate their activity or properties. The modeling studies confirmed that these conjugates bind in the active site of LpxC and provided further insight into which conjugates show the most promise as inhibitors of LpxC.

## 2. Results and Discussion

### 2.1. Computational

The triazole-linked conjugates were optimized in the LpxC active site using the M06-L [19] density functional theory methods with the 6-31G [20] basis set. The ligands were placed in a truncated active site (His265, Gly264, His58, Phe192, Phe194, Glu197, and Lys239) in an initial configuration similar to the natural substrate (from the crystal structure) [21]. The positions of the ligands and amino acid residue side chains were optimized in both a vacuum model and a solvated model. For the solvated model, we used implicit solvation with water via the PCM using default parameters [22]. Counterpoise-corrected [23] electronic interaction energies were calculated for the ligand and Zn^2+^ and the ligand and all amino acid residues in both conditions using M06-L and the 6-311+G* basis set.

The results of the counterpoise-corrected electronic interaction energies of compounds **1b**, **2b** and **3** in the vacuum model are shown in Table 1. Negative numbers indicate attractive forces. Conjugates **1b** and **2b** were evaluated as hydroxamic acids to ensure the strongest interaction with the zinc ion. Nonetheless, in the vacuum model, conjugate **3** shows the most favorable interaction energy in the active site, with a large percentage of the total interaction energy coming from the interaction between the ligand and the zinc ion. Compounds **1b** and **2b** were designed to have added flexibility to adopt the optimal conformation and binding within the LpxC active site. However, there is a possibility the compounds had too much rotational freedom, resulting in lower interaction energies. Due to the less favorable computational results of compounds **1b** and **2b**, they were not investigated further.

The results of the counterpoise-corrected interaction energies of the solvated model are summarized in the third and fourth columns of Table 1. The solvated model can be considered a more realistic model, as it is more representative of the conditions in vivo. Due to the favorability of compound **3** in the vacuum model, only **3** and **4** were investigated in the solvated model. It was concluded that **3** exhibits the strongest interaction energy because of the hydroxamic acid oxygen interacting with the backbone NH_2_ of the histidine residue. These computational studies suggest that **3** and **4** may act as inhibitors of LpxC, where a nitrogen in the triazole ring provides an additional point of interaction with zinc.

**Figure 3:**
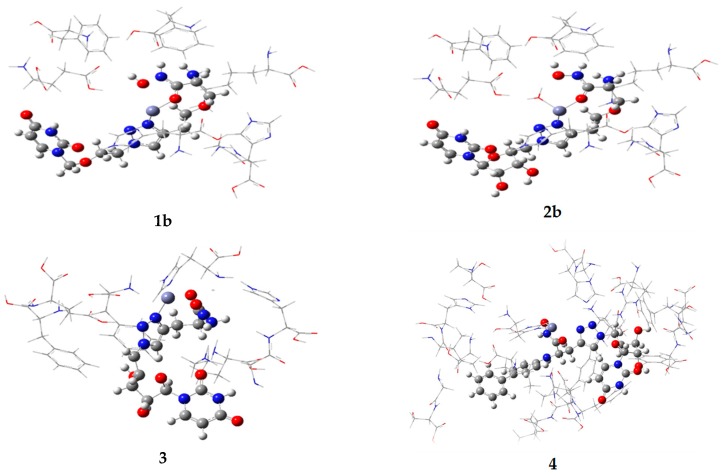
Optimized conformations of conjugates **1b**, **2b**, **3** and **4** in the truncated LpxC active site. The purple atom is Zn^2+^. Conjugates **1b** and **2b** were optimized in vacuo, while **3** and **4** were evaluated in the solvated model.

The optimized complexes are shown in Figure 3. Since the Zn^2+^ ion in the active site has a positive charge, it can bind with any portion of the substrate that is partially negative. The ligand binds Zn^2+^ using the oxygens of the hydroxamic acid, producing a very strong attractive interaction. However, our results showed the triazole group also binds well to zinc, which may strengthen binding in the active site. All four conjugates bound Zn^2+^ through the hydroxamic acid oxygens and with a nitrogen in the triazole ring. Lys239 and His58 have strong attractive interactions with conjugate **4**. This is because the ligand has a −1 charge and both lysine and histidine are positively charged. The glutamate residues in the active site have strong repulsive interactions with **4** since glutamate is negatively charged, as is compound **4**.

### 2.2. Synthesis

**Scheme 1:**
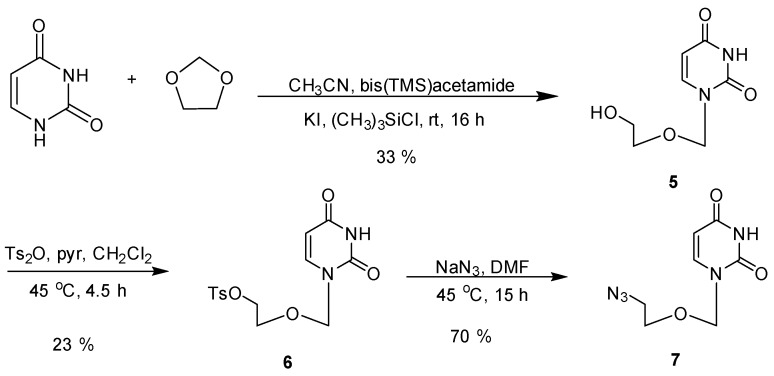
Synthesis of the azido acyclic nucleoside **7**.

To synthesize the acyclic nucleoside **5**, uracil was reacted with 1,3-dioxolane according to the published procedure [24] to produce **5** in a 33% yield, (Scheme 1). The acyclic nucleoside **5** was tosylated in **6**, followed by exchanging the tosylate for an azide **7** (Scheme 1) [25]. In our hands, tosylation of the acyclic nucleoside analogue proved to be difficult. Various attempts to synthesize **6** are depicted in Supplementary Table S1. A review of the literature for more successful methods was unproductive [25,26,27,28,29]. Both tosyl anhydride (Ts_2_O) or tosyl chloride (TsCl) have been used, as well as the presence or absence of an aqueous workup. Pure tosylate **6** was obtained with a yield of 23% and a clean ^1^H-NMR. The azide reaction proceeded with success to about 70% yield. Similar reactions have been perfomed resulting in higher yields [29]. However, differences in solubility and polarity of their 5,6-substituted uracil analogue likely played a role in the inconsistency in their reported yield and what we obtained.

**Scheme 2:**
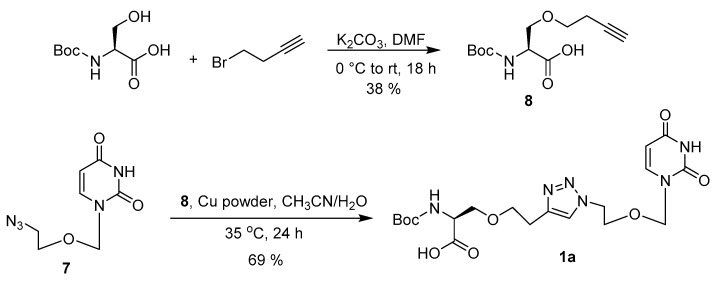
Synthesis of acyclic nucleoside conjugate **1a**.

Synthesis of the acyclic nucleoside-Ser conjugate is illustrated in Scheme 2. Commercially available Boc-l-Ser was alkylated with bromobutyne to produce **8** (Scheme 2). The alkylation reaction was first attempted using NaH as the base, producing **8** in 10–23% yields [30]. Due to the poor yields and inconsistent purity of the compound, a different method was sought. Using K_2_CO_3_ as a base, **8** was obtained in a yield of 38% [31].

CuAAC was used to create the 1,2,3-triazole linkage to couple the nucleoside analogue and amino acid. Azide **7** and alkyne **8** were coupled together to form **1a** [32]. Despite the difficulty in preparation of the azide and alkyne, the click reaction proceeded with ease, providing **1a** in a 69% yield. Silica gel column chromatography eluted a pure compound, despite the impurities present in the azide.

**Scheme 3:**
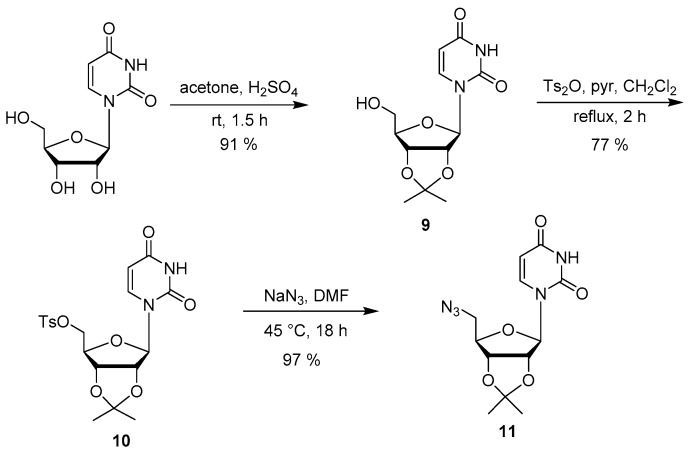
Synthesis of the azidonucleoside **11**.

The remaining conjugates **2**–**4** were synthesized from the protected 5′-azido uridine **11**, which was synthesized in three steps (Scheme 3). Beginning with a ketal protection of the 2′- and 3′-hydroxyl groups on commercially available uridine to produce **9** (Scheme 3) [33]. The ketal production proceeded with ease, attaining a high yield of 91%. Following the protection, a tosylation reaction was performed to activate the 5′-hydroxyl group, producing **11** (Scheme 3) [25]. Once again, the tosylation reaction proceeded with some difficulty. There are literature procedures describing the use of either Ts_2_O or TsCl, both of which have been used with pyridine, as well as in the presence or absence of dichloromethane [25,34]. Various reaction conditions were attempted to determine the optimal conditions to produce a pure product in a high yield, including running the reaction for 2–4 h or overnight, at room temperature or slightly heated. Despite the literature [25] reporting a 98% yield on the formation of the tosylate, in our hands a maximum of 77% yield has been obtained with Ts_2_O and dichloromethane under a 2 h reflux. The tosyl group in **11** was replaced by an azide group in accordance with the literature procedure [25] (Scheme 3). The yields for this reaction are comparable to the literature.

**Scheme 4:**
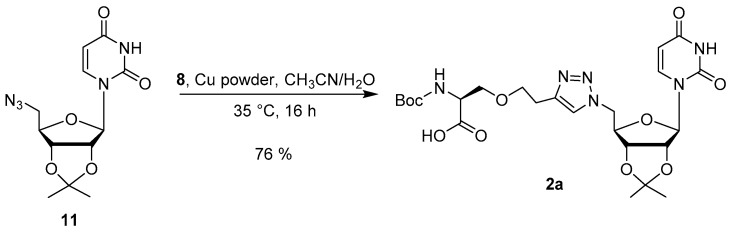
CuAAC of **8** and **11**.

Alkyne **8** and azide **11** were clicked together to form the uridine-Ser conjugate **2a** as shown in Scheme 4. Initially the reaction ran 48 h with a copper wire-wrapped stir bar to provide product **2a** in 45% yield [32]. However, when Cu powder was used, the reaction yield improved significantly to 76%. Due to the synthetic challenges observed in the preparation of **1a** and **2a**, further deprotection and conversion to the hydroxamic acid was not pursued.

Following the challenges with alkylating Boc-l-Ser, we envisioned the use of propargylglycine to bypass the need for the alkylation reaction. Therefore, commercially available l-propargylglycine was used as the amino acid to click to the nucleoside. The use of propargylglycine provides significant advantages in that it allows further functionalization of the amino acid, including the convenient coupling of another amino acid or any other pharmacophore on either the nitrogen or carboxylic acid and the conversion to a hydroxamic acid or ester.

**Scheme 5:**
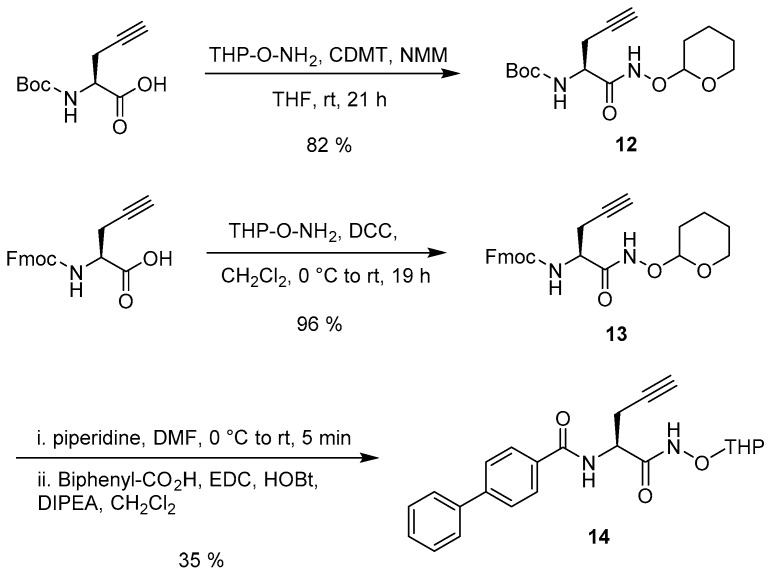
Preparation of alkynes **12** and **14**.

The synthesis of the uridine-propargylglycine conjugates began with the conversion of the carboxylic acid of l-propargylglycine to the protected hydroxamic acid intermediates **12** and **13**. Compound **12** was successfully synthesized with an 82% yield using commercially available Boc-l-propargylglycine and *O*-(tetrahydro-2H-pyran-2-yl)hydroxylamine (THP-hydroxylamine), 2-chloro-4,6-dimethoxy-1,3,5-triazine (CDMT) and N-methylmorpholine (NMM) (Scheme 5) [35]. Commercially available Fmoc-l-propargylglycine was initially converted to the protected hydroxamic acid **13** using CDMT and NMM [35]. However, when standard dicyclohexylcarbodiimide (DCC) coupling was used, purification was easier and higher yields were obtained. Next, the Fmoc protecting group was removed using a 20% solution of piperidine in dimethylformamide (DMF), and immediately coupled with biphenyl-4-carboxylic acid using 1-ethyl-3-(3-dimethylaminopropyl)carbodiimide (EDC) to produce **14** in a 35% yield over the two steps (Scheme 5) [36]. Not surprisingly, the use of diethylamine and longer reaction times during the Fmoc deprotection resulted in lower yields and racemization.

**Scheme 6:**
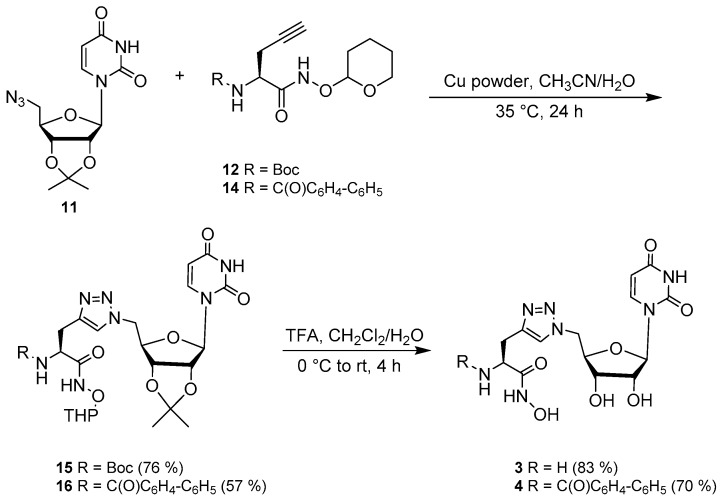
Synthesis of compounds **3** and **4**.

Following preparation of the appropriate alkynes **12** and **14**, they were coupled to azide **11** via a Cu-catalyzed cycloaddition [32] (Scheme 6). The click reaction was first sonicated for 10–15 min before stirring overnight and proceeded in 76 and 57% yields. Finally, the Boc group was removed (**3**) and the 2′- and 3′-hydroxyl groups were deprotected with trifluoroacetic acid (TFA) to yield **3** and **4** in an 83% and 70% yield, respectively (Scheme 6). The final products were precipitated from a methanolic solution using diethyl ether. The final conjugates **3** and **4** were characterized by ^1^H- and ^13^C-NMR and HRMS. The purity was confirmed by HPLC.

**Figure 4:**
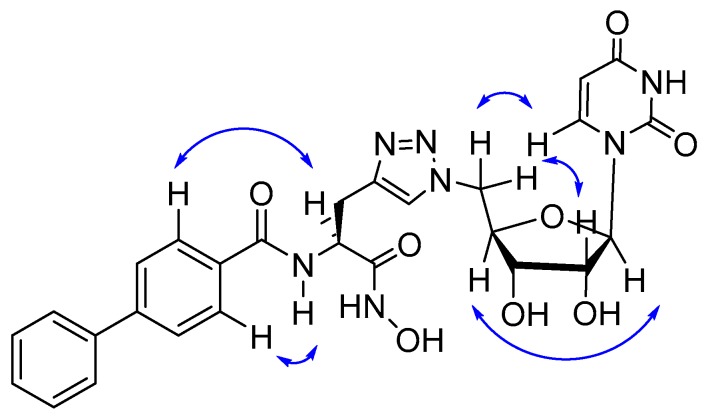
Key NOESY correlations of **4**.

The structure of **4** was also confirmed by ^1^H-^1^H COSY and 2D-NOESY experiments (Supplementary Figures S1 and S2). NOE correlations were observed between H-6 and H-5′, H-2′ and H-5′, H-1′ and H-4′, and H-α and H-2′′ and H-N and H-2′′ (Figure 4). Interestingly, there were no correlations observed for the amino acid methylene protons. Although some of the ^1^H signals are overlapping, the NOESY crosspeaks confirmed the presence of the expected 1,4-disubstituted-1,2,3-triazole.

## 3. Materials and Methods

### 3.1. General Methods

Unless otherwise indicated, all anhydrous solvents were commercially obtained and stored in Sure-Seal bottles under argon. All other reagents and solvents were purchased as the highest grade available from Acros (Fisher Scientific, Pittsburgh, PA, USA) or Sigma-Aldrich (St. Louis, MO, USA) and were used without further purification. Boc-l-Propargylglycine was purchased from Aurum Pharmatech (Franklin Park, NJ, USA) and Fmoc-l-propargylglycine was purchased from AK Scientific (Union City, CA, USA). All moisture-sensitive reactions were carried out using dry solvents and under slight pressure of ultra-pure argon. Commercially available disposable syringes were used for transferring reagents and solvents. All single syntheses were conducted in conventional flasks under an atmosphere of dry argon. Proton (^1^H) and carbon (^13^C) NMR spectra were recorded on a 400 MHz spectrometer (Varian, Palo Alto, CA USA). NMR data are reported as follows: chemical shifts (δ) are reported in parts per million (ppm) referenced to ^1^H (CDCl_3_ at 7.27, CD_3_OD at 3.31, DMSO-*d*_6_ at 2.50), ^13^C (CDCl_3_ at 77.16, CD_3_OD at 49.00, DMSO-*d*_6_ at 39.52), multiplicity (s = singlet, br s = broad singlet, d = doublet, dd = doublet of doublets, t = triplet, q = quartet, quin = quintet, m = multiplet) and coupling constants (*J*) are reported in Hz. Column chromatography was conducted using silica gel (Silicycle 55–65 Å). Purity of the compounds was confirmed to be greater than 95% via HPLC analysis (LC-6AD pumps, detection with a SPD-M20A PDA and CBM-20A communication, Shimadzu, Columbia, MD, USA) and a Hypersil Gold C18 column, (250 × 4.6 mm, particle size = 5 μm, Thermo Fisher Scientific, Waltham, MA, USA). High resolution mass spectra (HRMS) were obtained in the University of California Riverside High Resolution Mass Spectrometry facility using +ESI.

### 3.2. Computational Methods

Starting from the LpxC crystal structure with the natural substrate bound in the active site (PDB ID:2IER) [21], proposed analogues were placed in the active site in a similar configuration to the natural substrate. Optimizations were completed using M06-L/6-31G. M06-L was used since it is effective for intermolecular forces and interactions with metals [19]. A relaxed active site where the heavy atoms on the amino acid side chains and all protons were allowed to adjust their positions was used. Optimizations with implicit solvation were done via the PCM model using default parameters. The solvent in these cases was water. Interaction energies were also calculated from the solvent optimized structures. In all cases, counterpoise corrected interaction energies were calculated for the ligand/amino acid pairs and ligand/Zn^2+^ using M06-L/6-311+G*. Calculations were performed using Gaussian09 [37].

### 3.3. Synthesis of Acyclic Nucleoside Conjugate ***1***

*1-((2-Hydroxyethoxy)methyl)pyrimidine-2,4(1H,3H)-dione* (**5**). To a flask containing uracil (1.51 g, 13.5 mmol) was added CH_3_CN (34 mL, 0.4 mmol/mL) to form a suspension under Ar. At room temperature (r.t.), bis(TMS)acetamide (9.25 mL, 37.8 mmol) was added slowly. After 1 h at r.t., 1,3-dioxolane (1.0 mL, 14.3 mmol), KI (2.22 g, 13.4 mmol) and TMSCl (2.4 mL, 18.9 mmol) were added to the clear solution at r.t. After 16 h, the yellow, cloudy reaction mixture was quenched with MeOH (25 mL). The mixture turned white and was then neutralized with NaHCO_3_ (5.12 g, added in three portions, 2 min apart). The solid was removed by filtration and the filtrate was concentrated under reduced pressure. The crude product was purified by silica gel column chromatography (EtOAc:MeOH, 10–20% *v*/*v*) The crystals formed in fractions were collected, while the liquid from the fractions was concentrated under reduced pressure. The non-crystalline solid was precipitated out with isopropanol, yielding **5** as an off-white solid (835 mg, 33%). *R*_f_ (4:1 EtOAc–MeOH) = 0.44. ^1^H-NMR (DMSO-*d*_6_): δ 3.46–3.50 (m, 4H) 5.08 (s, 2H) 5.61 (d, *J* = 7.8 Hz, 1H) 7.70 (d, *J* = 7.8 Hz, 1H) 11.34 (s, 1H). The ^1^H-NMR data was similar to the literature values [38]. 

*2-((2,4-Dioxo-3,4-dihydropyrimidin-1(2H)-yl)methoxy)ethyl 4-methylbenzenesulfonate* (**6**). Compound **5** (230 mg, 1.24 mmol) was co-evaporated with pyridine (3 × 2 mL) under Ar and was then redissolved in pyridine (3.1 mL, 0.39 mmol/mL) under Ar and Ts_2_O (640 mg, 1.96 mmol) was added at 0 °C. The reaction ran for 4.5 h at 30 °C. After 4.5 h, the reaction mixture was diluted with CH_2_Cl_2_ and washed with 0.5 M HCl (4 × 7 mL), brine (3 × 3 mL), and saturated NaHCO_3_ solution (2 × 7 mL). The organic layer was collected, dried with Na_2_SO_4_, and concentrated under reduced pressure. The crude product was purified by silica gel column chromatography (CH_2_Cl_2_:MeOH, 10%). The reaction afforded the desired product **6** (95 mg, 23%) as a yellow solid. *R*_f_ (1:4:0.5 Hex–EtOAc–MeOH) = 0.48. ^1^H-NMR (CHCl_3_-*d*): δ 2.41 (s, 3H) 3.76 (t, *J* = 4.4 Hz, 2H) 4.11 (t, *J* = 4.4 Hz, 2H) 5.10 (s, 2H) 5.72 (d, *J* = 7.0 Hz, 1H) 7.25 (d, *J* = 7.8 Hz, 1H) 7.31 (d, *J* = 7.8 Hz, 2H) 7.73 (d, *J* = 7.0 Hz, 2H) 9.84 (s, 1H). ^13^C-NMR (CDCl_3_): δ 21.7, 67.3, 68.6, 76.8, 103.4, 127.9, 129.9, 132.6, 143.3, 145.1, 151.3, 163.8. The ^1^H-NMR data was similar to the literature values [38].

*1-((2-Azidoethoxy)methyl)pyrimidine-2,4(1H,3H)-dione* (**7**). Tosylate **6** (253 mg, 0.74 mmol) was dissolved in DMF (1.8 mL) under Ar. NaN_3_ (268 mg, 4.12 mmol) was added to the solution. The reaction was stirred for 15 h at 45 °C. After 15 h, the reaction was concentrated under reduced pressure. The crude product was purified by silica gel column chromatography (CH_2_Cl_2_:MeOH, 10%) to afford product **7** (268 mg, 70%) as a white solid. *R*_f_ (9:1 CH_2_Cl_2_–MeOH) = 0.74. ^1^H-NMR (CDCl_3_): δ 3.40–3.44 (m, 5H) 5.18 (m, 2H) 5.75 (dt, *J* = 7.9, 1.6 Hz, 1H) 7.32 (d, *J* = 7.9 Hz, 1H). The ^1^H-NMR data was similar to the literature values [38]. 

*O-(But-3-yn-1-yl)-N-(tert-butoxycarbonyl)-l-serine* (**8**). Boc-l-Ser-OH (246 mg, 1.20 mmol) and K_2_CO_3_ (348 mg, 2.52 mmol) were dissolved in anhydrous DMF (6.5 mL) under Ar. 4-Bromo-1-butyne (0.1 mL, 1.07 mmol) was added dropwise. The reaction mixture was heated to 45 °C and stirred overnight. The reaction mixture was poured over ice cold 2 M HCl, and the crude product was extracted with EtOAc (2 × 15 mL) and the combined organic extract was washed with 2 M HCl (2 × 15 mL) and brine (2 × 15 mL), concentrated and purified by silica gel column chromatography (Hex:EtOAc, 1:4, 1:3, 1:2) to yield alkyne **8** as a pale yellow oil (99 mg, 38%). *R*_f_ (1:4 Hex–EtOAc) = 0.75. ^1^H-NMR (CDCl_3_): δ 5.47 (br s, 1H), 4.34 (br s, 1H), 4.30–4.23 (m, 1H), 4.22–4.14 (m, 1H), 3.94 (dd, *J* = 3.5, 11.3 Hz, 1H), 3.83 (dd, *J* = 3.5, 11.3 Hz, 1H), 2.55–2.48 (m, 2H), 1.97 (t, *J* = 2.7 Hz, 1H), 1.39 (s, 9H). ^13^C-NMR (CDCl_3_): δ 18.7, 28.2, 55.7, 62.9, 70.2, 79.7, 155.7, 170.7, 173.0.

*N-(tert-Butoxycarbonyl)-O-(2-(1-(2-((2,4-dioxo-3,4-dihydropyrimidin-1(2H)-yl)methoxy)ethyl)-1H-1,2,3-triazol-4-yl)ethyl)-l-serine* (**1a**). In a 5 mL round bottom flask, azide **7** (103 mg, 0.49 mmol) was added to alkyne **8** (92 mg, 0.36 mmol) dissolved in CH_3_CN (0.7 mL). The alkyne container was rinsed with CH_3_CN (0.5 mL) and added to the reaction flask. To the flask was added DI water (0.4 mL, 1.74 mmol/mL) and Cu powder (9 mg, 0.14 mmol). The reaction was ultra-sonicated for 10 min before being stirred at 35 °C overnight. At 21 h, a second addition of Cu powder (9 mg, 0.14 mmol) was added. The reaction flask was ultra-sonicated for 10 min and stirred at 35 °C. At 24 h, the reaction was concentrated under reduced pressure. The crude product was purified by silica gel column chromatography (CH_2_Cl_2_:MeOH, 5%) to afford the desired product **1** as a white solid (112 mg, 69%). *R*_f_ (9:1 CH_2_Cl_2_–MeOH) = 0.38. ^1^H-NMR (CDCl_3_): δ 1.43 (s, 9H) 2.95–3.17 (m, 2H) 3.79 (m, 1H) 4.01 (t, *J* = 4.8 Hz, 2H) 4.15–4.27 (m, 1H) 4.35 (d, *J* = 8.1 Hz, 1H) 4.52 (t, *J* = 4.8 Hz, 2H) 4.56–4.66 (m, 1H) 4.76 (s, 1H) 5.11 (s, 2H) 5.30 (s, 2H) 5.69–5.78 (m, 2H) 7.25 (dd, *J* = 8.0, 1.2 Hz, 1H) 7.57 (s, 1H) 9.81 (s, 1H). ^13^C-NMR (CDCl_3_): δ 25.0, 28.3, 49.9, 53.5, 56.1, 62.8, 64.1, 67.7, 79.9, 103.2, 123.02, 143.69, 144.19, 151.43, 155.79, 163.92, 171.15.

### 3.4. Synthesis of the Uridine-Amino Acid Conjugates ***2***–***4***

*1-((3aR,4R,6R,6aR)-6-(Hydroxymethyl)-2,2-dimethyltetrahydrofuro[3,4-d][1,3]dioxol-4-yl)pyrimidine-2,4(1H,3H)-dione* (**9**). Uridine (1.50 g, 6.14 mmol) was stirred in acetone (75 mL, 0.08 mmol/mL) and treated with H_2_SO_4_ (0.75 mL) dropwise at r.t. and stirring at r.t. was continued. After 1.5 h, the reaction was neutralized with Et_3_N (2.25 mL) and concentrated under reduced pressure. The crude mixture was purified by silica gel column chromatography (CH_2_Cl_2_:MeOH, 0–8%) to afford alcohol **9** (1.62 g, 93%) as a white solid. *R*_f_ (9:1 CH_2_Cl_2_–MeOH) = 0.33. ^1^H-NMR (CDCl_3_): δ 1.36 (s, 3H) 1.58 (s, 3H) 3.81–3.92 (m, 3H) 4.29 (q, *J* = 3.4 Hz, 1H) 4.96 (dd, *J* = 6.4, 3.4 Hz, 1H) 5.04 (dd, *J* = 6.4, 2.9 Hz, 1H) 5.61 (d, *J* = 2.9 Hz, 1H) 5.74 (d, *J* = 8.1 Hz, 1H) 7.41 (d, *J* = 8.0 Hz, 1H) 9.42 (s, 1H). ^13^C-NMR (CDCl_3_): δ 25.2, 27.2, 50.6, 62.4, 80.2, 80.4, 83.7, 86.8, 102.4, 114.3, 142.7, 150.4. The NMR data was similar to the literature values [25].

*((3aR,4R,6R,6aR)-6-(2,4-Dioxo-3,4-dihydropyrimidin-1(2H)-yl)-2,2-dimethyltetrahydrofuro[3,4-d][1,3]-dioxol-4-yl)methyl 4-methylbenzenesulfonate* (**10**). Alcohol **9** (0.90 g, 3.17 mmol) was dissolved in anhydrous CH_2_Cl_2_ (14 mL) under Ar and pyridine (2.7 mL) was added. To the mixture was added Ts_2_O (1.71 g, 5.25 mmol). The light brown solution was heated under reflux for 2 h. After 2 h, the solution was diluted with CHCl_3_ (50 mL) and washed with 0.5 M HCl (5 × 22 mL) and saturated NaHCO_3_ solution (2 × 22 mL). The organic layer was dried over Na_2_SO_4_, filtered, and concentrated under reduced pressure. The crude product was purified by silica gel column chromatography (CHCl_3_:MeOH, 0–2%) to afford tosylate **10** (1.06 g, 77%) as a red foam. *R*_f_ (CH_2_Cl_2_–MeOH, 2%) = 0.17. ^1^H-NMR (CDCl_3_): δ 1.31 (s, 3H) 1.52 (s, 3H) 2.41 (s, 3H) 4.22–4.29 (m, 2H) 4.29–4.35 (m, 1H) 4.78 (dd, *J* = 6.4, 3.8 Hz, 1H) 4.94 (dd, *J* = 6.4, 2.0 Hz, 1H) 5.63 (d, *J* = 2.0 Hz, 1H) 5.71 (d, *J* = 8.1 Hz, 1H) 7.24 (d, *J* = 8.1 Hz, 1H) 7.31 (d, *J* = 8.0 Hz, 2H) 7.74 (d, *J* = 8.1 Hz, 2H) 9.89 (s, 1H). The NMR data was similar to the literature values [25].

*1-((3aR,4R,6R,6aR)-6-(Azidomethyl)-2,2-dimethyltetrahydrofuro[3,4-d][1,3]dioxol-4-yl)pyrimidine-2,4-(1H,3H)-dione* (**11**). Tosylate **10** (153 mg, 0.35 mmol) was dissolved in DMF (1 mL) under Ar. NaN_3_ (110 mg, 1.70 mmol) was added to the solution. The reaction ran for 2 days (1st day at rt, 2nd day at 45 °C). The reaction mixture was concentrated under reduced pressure. The crude product was purified by silica gel column chromatography (CH_2_Cl_2_:MeOH, 0–20%) to afford azide **11** (103 mg, 96%) as a white solid. *R*_f_ (2:3 Hex–EtOAc) = 0.36. ^1^H-NMR (CDCl_3_): δ 1.36 (s, 3H) 1.57 (s, 3H) 3.63 (d, *J* = 5.2 Hz, 2H) 4.24 (q, *J* = 5.0 Hz, 1H) 4.82 (dd, *J* = 6.5, 4.2 Hz, 1H) 5.01 (dd, *J* = 6.5, 2.1 Hz, 1H) 5.66 (d, *J* = 2.1 Hz, 1H) 5.77 (d, *J* = 8.0, 1.4 Hz, 1H) 7.30 (d, *J* = 8.1 Hz, 1H) 9.50 (s, 1H). The NMR data was similar to the literature values [25].

*N-(tert-Butoxycarbonyl)-O-(2-(1-(((3aR,4R,6R,6aR)-6-(2,4-dioxo-3,4-dihydropyrimidin-1(2H)-yl)-2,2-dimethyltetrahydrofuro[3,4-d][1,3]dioxol-4-yl)methyl)-1H-1,2,3-triazol-4-yl)ethyl)-l-serine* (**2a**). Azide **11** (110 mg, 0.43 mmol) was dissolved in CH_3_CN (1.5 mL) and added to alkyne **8** (135 mg, 0.44 mmol). DI H_2_O (0.5 mL) was added to the reaction flask along with Cu powder (5 mg, 0.08 mmol). The reaction was sonicated for 10 min before stirring at 35 °C overnight. The reaction was monitored by TLC (2:3 Hex:EtOAc). At 16 h, the reaction was concentrated under reduced pressure, and the crude product (blue-green foam) was purified by silica gel column chromatography (CH_2_Cl_2_–MeOH, 2–5%) to afford product **2** (207 mg, 76%) as a white solid. *R*_f_ (9:1 CH_2_Cl_2_–MeOH) = 0.60. ^1^H-NMR (CDCl_3_): δ 10.04 (br s, 1H), 7.46 (s, 1H), 7.14 (d, *J* = 8.2 Hz, 1H), 5.68 (d, *J* = 8.2 Hz, 2H), 5.44 (d, *J* = 1.2 Hz, 1H), 5.11–4.98 (m, 1H), 4.86 (dd, *J* = 4.3, 6.3 Hz, 1H), 4.70–4.51 (m, 3H), 4.46–4.37 (m, 1H), 4.27 (d, *J* = 8.6 Hz, 1H), 4.05 (q, *J* = 7.0 Hz, 4H), 1.98 (s, 3H), 1.47 (s, 3H), 1.42–1.32 (m, 9H), 1.27 (s, 3H), 1.22–1.16 (m, 3H).

*tert-Butyl ((2S)-1-oxo-1-(((tetrahydro-2H-pyran-2-yl)oxy)amino)pent-4-yn-2-yl)carbamate* (**12**). Boc-l-propargylglycine (584 mg, 2.74 mmol) was measured out in a glove bag under an atmosphere of Ar. To the alkyne was added CDMT (484 mg, 2.76 mmol) and anhydrous THF (19 mL). At r.t., NMM (0.35 mL, 3.18 mmol, 1.16 eq) was added and the reaction mixture was stirred. After 1.5 h, THP-*O*-NH_2_ (373 mg, 3.18 mmol) was added. After 19.5 h, the reaction mixture was filtered through Celite (~2 inches). The Celite was rinsed with CH_2_Cl_2_. The resulting solution was concentrated and purified by silica gel column chromatography (Hex:EtOAc 1:4) to afford **12** (761 mg, 89%). *R*_f_ (1:4 Hex–EtOAc) = 0.73. ^1^H-NMR (CDCl_3_): δ 1.38 (s, 9H) 1.46–1.63 (m, 4H) 1.68–1.83 (m, 4H) 2.60 (s, 2H) 3.55 (m, 1H) 3.91 (s, 2H) 4.28 (t, *J* = 7.0 Hz, 1H) 4.92 (d, *J* = 2.5 Hz, 1H) 5.54 (d, *J* = 7.7 Hz, 1H) 9.96 (s, 1H). ^13^C-NMR (CDCl_3_): δ 167.6, 102.3, 80.3, 79.1, 77.1, 71.5, 62.3, 54.8, 50.8, 24.9, 22.6, 20.9, 18.5.

*(9H-Fluoren-9-yl)methyl (1-oxo-1-(((tetrahydro-2H-pyran-2-yl)oxy)amino)pent-4-yn-2-yl)carbamate* (**13**). Fmoc-l-propargylglycine (511 mg, 1.49 mmol) and THP-*O*-NH_2_ (219 mg, 1.64 mmol) were dissolved in anhydrous CH_2_Cl_2_ (2.5 mL) under Ar gas. In a separate flask, DCC (340 mg, 1.64 mmol) was dissolved in anhydrous CH_2_Cl_2_ (3.5 mL) under Ar gas and transferred dropwise to the solution containing Fmoc-l-propargylglycine and THP-*O*-NH_2_ at 0 °C. The reaction mixture was then warmed to r.t. and ran for 19 h. The crude mixture was filtered, concentrated under reduced pressure, and purified by silica gel column chromatography (CH_2_Cl_2_:MeOH, 5%) to afford **13** (642 mg, 96%) as a white solid. *R*_f_ (CH_2_Cl_2_–MeOH, 5%) = 0.66. ^1^H-NMR (CDCl_3_): δ 9.99 (br s, 1H), 7.73 (d, *J* = 7.4 Hz, 2H), 7.56 (d, *J* = 7.0 Hz, 2H), 7.37 (t, *J* = 7.5 Hz, 2H), 7.27 (t, *J* = 7.5 Hz, 2H), 5.99 (d, *J* = 7.8 Hz, 1H), 5.27 (s, 2H), 4.95 (br s, 1H), 4.54–4.26 (m, 3H), 4.18 (t, *J* = 7.0 Hz, 1H), 3.88 (t, *J* = 10.0 Hz, 1H), 3.52 (d, *J* = 11.1 Hz, 1H), 2.69 (br s, 2H), 2.10–2.02 (m, 1H), 1.85–1.70 (m, 2H), 1.63–1.42 (m, 3H).

*N-(1-Oxo-1-(((tetrahydro-2H-pyran-2-yl)oxy)amino)pent-4-yn-2-yl)-[1,1'-biphenyl]-4-carboxamide* (**14**). Compound **13** (759 mg, 1.75 mmol) was dissolved in anhydrous CH_2_Cl_2_ (3.5 mL) under Ar and the solution was cooled to 0 °C. Piperidine (20% in DMF, 2 mL) was added dropwise to the solution. After the reaction was complete, as determined by TLC, the reaction mixture was concentrated under pressure. Immediately following the deprotection reaction, the crude product, biphenyl-4-carboxylic acid (285 mg, 1.44 mmol), EDC·HCl (409 mg, 2.13 mmol), and HOBt (303 mg, 1.98 mmol) were dissolved in anhydrous CH_2_Cl_2_ (15 mL) under Ar gas at 0 °C. Next, DIPEA (1 mL, 5.74 mmol) was added to the reaction mixture at 0 °C. The reaction ran for 1 h at 0 °C and then warmed to r.t. to run for 18 h. After stopping the reaction, the reaction mixture was diluted with CH_2_Cl_2_ (15 mL) and washed with 1.6 M citric acid (pH ~ 3, 30 mL), saturated NaHCO_3_ solution (30 mL), and brine (30 mL). The organic layer was dried with Na_2_SO_4_, filtered, and concentrated under reduce pressure. The crude product was then purified via silica gel column chromatography (CH_2_Cl_2_:MeOH, 5%, Hex:EtOAc, 1:1) to produce **14** (240 mg, 35%) as a white solid. *R*_f_ (1:1 Hex–EtOAc) = 0.44. ^1^H-NMR (CDCl_3_): δ 7.88 (m, 2H), 7.67–7.51 (m, 4H), 7.48–7.30 (m, 4H), 5.09–4.98 (m, 1H), 4.92 (t, *J* = 6.7 Hz, 1H), 4.16–4.05 (m, 1H), 3.96 (t, *J* = 9.0 Hz, 1H), 3.67–3.50 (m, 1H), 2.96–2.77 (m, 2H), 2.22–2.15 (m, 1H), 2.11 (td, *J* = 2.6, 5.4 Hz, 1H), 2.05–2.00 (m, 2H), 1.95–1.71 (m, 4H), 1.71–1.60 (m, 2H), 1.60–1.46 (m, 3H), 1.27–1.20 (m, 2H), 1.15–1.03 (m, 1H).

*tert-Butyl ((2S)-3-(1-(((3aR,4R,6R,6aR)-6-(2,4-dioxo-3,4-dihydropyrimidin-1(2H)-yl)-2,2-dimethyltetra-hydrofuro[3,4-d][1,3]dioxol-4-yl)methyl)-1H-1,2,3-triazol-4-yl)-1-oxo-1-(((tetrahydro-2H-pyran-2-yl)oxy)-amino)propan-2-yl)carbamate* (**15**). Azide **11** (155 mg, 0.501 mmol) was dissolved in CH_3_CN (3.4 mL) and added to alkyne **12** (149 mg, 0.477 mmol). DI water (0.6 mL) and Cu powder (13 mg, 0.21 mmol) were added. The reaction was sonicated for 10 min before stirring at 35 °C overnight. At 21 h, the reaction was concentrated under reduced pressure. The green crude product was purified by silica gel column chromatography (CH_2_Cl_2_:MeOH 6%) to afford the desired product **15** (225 mg, 76%) as an off-white solid. *R*_f_ (CH_2_Cl_2_–MeOH, 6%) = 0.44. ^1^H-NMR (CDCl_3_): δ 1.30 (s, 3H) 1.37 (s, 8H) 1.50 (s, 6H) 1.72 (s, 3H) 3.12 (s, 1H) 3.54 (s, 1H) 3.91 (m, 1H) 4.41 (s, 1H) 4.67 (s, 1H) 4.86 (s, 1H) 5.02 (s, 1H) 5.54 (d, *J* = 10.2 Hz, 1H) 5.71 (s, 1H) 5.82 (s, 1H) 7.16 (s, 1H) 7.43–7.66 (m, 1H) 10.24–10.47 (m, 1H).

*N-(3-(1-(((3aR,4R,6R,6aR)-6-(2,4-Dioxo-3,4-dihydropyrimidin-1(2H)-yl)-2,2-dimethyltetrahydrofuro[3,4-d][1,3]dioxol-4-yl)methyl)-1H-1,2,3-triazol-4-yl)-1-oxo-1-(((tetrahydro-2H-pyran-2-yl)oxy)amino)propan-2-yl)-[1,1'-biphenyl]-4-carboxamide* (**16**). Azide **11** (179 mg, 0.58 mmol) was first dissolved in CH_3_CN (1.25 mL), and the solution was transferred to a flask containing **14** (240 mg, 0.61 mmol). Upon dissolution of **14**, Cu powder (13 mg, 0.20 mmol) and DI H_2_O (1.0 mL) were added to the reaction mixture. The reaction mixture was then subjected to sonication for 15 min, and then stirred for 21 h at 35 °C. The crude reaction mixture was concentrated under reduced pressure and purified via silica gel column chromatography (CH_2_Cl_2_:MeOH, 5%) to yield **16** (231 mg, 57%) as a white solid. *R*_f_ (CH_2_Cl_2_–MeOH, 5%) = 0.32. ^1^H-NMR (CDCl_3_): δ 7.87 (d, *J* = 8.2 Hz, 2H), 7.64–7.52 (m, 5H), 7.46–7.39 (m, 2H), 7.36 (d, *J* = 7.4 Hz, 1H), 7.11 (dd, *J* = 3.3, 8.0 Hz, 1H), 5.70 (d, *J* = 7.8 Hz, 1H), 5.47 (d, *J* = 3.9 Hz, 1H), 5.29 (s, 3H), 5.01 (t, *J* = 5.3 Hz, 1H), 4.98–4.89 (m, 2H), 4.87–4.79 (m, 1H), 4.66 (d, *J* = 5.1 Hz, 2H), 4.45–4.36 (m, 1H), 3.93 (br s, 1H), 3.55 (br s, 1H), 3.37 (d, *J* = 6.7 Hz, 1H), 3.24 (br s, 1H), 1.74 (br s, 2H), 1.49 (s, 3H), 1.26 (s, 3H).

*(S)-2-Amino-3-(1-(((2R,3S,4R,5R)-5-(2,4-dioxo-3,4-dihydropyrimidin-1(2H)-yl)-3,4-dihydroxytetrahydro-furan-2-yl)methyl)-1H-1,2,3-triazol-4-yl)-N-hydroxypropanamide* (**3**). Trifluoroacetic acid (3 mL, 90% in H_2_O) was added to **15** (187 mg, 0.30 mmol) dissolved in CH_2_Cl_2_ (1 mL) at 0 °C. After 14.5 h, the reaction was stopped and concentrated. The residue was rinsed with CH_2_Cl_2_ and concentrated followed by being transferred to a pear-flask in MeOH. The residue was dissolved in MeOH and precipitated out with Et_2_O to yield product **3** as an off-white solid (99 mg, 65%).^1^H-NMR (DMSO-*d*_6_): δ 9.23 (s, 1H), 7.80 (s, 1H), 7.50 (d, *J* = 8.0 Hz, 1H), 5.67 (d, *J* = 5.2 Hz, 1H), 5.60 (d, *J* = 8.0 Hz, 1H), 5.50 (d, *J* = 5.5 Hz, 1H), 5.35 (d, *J* = 5.5 Hz, 1H), 4.05 (quin, *J* = 5.2 Hz, 2H), 3.90 (q, *J* = 5.2 Hz, 1H), 3.78 (t, *J* = 7.0 Hz, 1H), 1.11–0.93 (m, 4H). ^13^C-NMR (DMSO-*d*_6_): δ 27.8, 50.6, 51.7, 65.4, 70.9, 72.5, 82.0, 89.5, 102.6, 124.7, 140.9, 141.7, 151.1, 163.4. HRMS (ESI) *m/z* calcd for [C_14_H_20_N_7_O_7_^+^] 398.1419 found 398.1423.

*N-(3-(1-(((2R,3S,4R,5R)-5-(2,4-Dioxo-3,4-dihydropyrimidin-1(2H)-yl)-3,4-dihydroxytetrahydrofuran-2-yl)methyl)-1H-1,2,3-triazol-4-yl)-1-(hydroxyamino)-1-oxopropan-2-yl)-[1,1'-biphenyl]-4-carboxamide* (**4**). For the deprotection reaction, **16** (222 mg, 0.32 mmol) was dissolved in CH_2_Cl_2_ at 0 °C, to which DI H_2_O (0.40 mL) and TFA (1 mL) were added dropwise. The reaction mixture warmed to r.t. and stirred for 20 h. The crude reaction mixture was concentrated under reduced pressure and dissolved in 1:1 CH_2_Cl_2_:MeOH (2 mL). The solution was transferred to a conical vial and concentrated under reduced pressure. Finally, the product was precipitated from a methanolic solution with Et_2_O to afford **4** (128 mg, 70%) as an off-white powder. ^1^H-NMR (DMSO-*d*_6_): δ 11.32 (br s, 1H), 8.74 (d, *J* = 7.8 Hz, 1H), 7.92–7.80 (m, 3H), 7.68 (d, *J* = 7.8 Hz, 2H), 7.72 (d, *J* = 8.2 Hz, 2H), 7.53–7.40 (m, 3H), 7.40–7.32 (m, 1H), 5.77–5.66 (m, 1H), 5.59 (d, *J* = 7.8 Hz, 1H), 4.74–4.53 (m, 3H), 4.07 (br s, 1H), 4.04–3.97 (m, 1H), 3.90 (t, *J* = 5.3 Hz, 1H), 3.29–3.07 (m, 2H). ^13^C-NMR (DMSO-*d*_6_): δ 27.4, 51.5, 53.1, 65.4, 70.9, 72.6, 82.2, 89.2, 102.6, 124.7, 126.9, 127.3, 128.5, 129.5, 133.2, 139.6, 141.4, 143.4, 143.8, 151.1, 163.4, 164.6. HRMS (ESI) *m*/*z* calcd for [C_27_H_28_N_7_O_8_^+^] 578.1994 found 578.2012.

## 4. Conclusions

This is the first report of nucleoside analogue-amino acid conjugates linked via a 1,2,3-triazole group. Using computational methods, the electronic interaction energies of conjugates **1b**, **2b**, **3** and **4** were determined in the LpxC active site. In the more realistic solvated model, compound **3** showed the most favorable interaction energy. The modeling studies suggest that conjugates **3** and **4** have the most potential as inhibitors of LpxC. Despite less favorable interaction energies, compounds **1a** and **2a** were synthesized and characterized by ^1^H-NMR. However, due to challenges in the synthesis of conjugates **1a** and **2a**, particularly the alkylation of serine to afford the alkyne for the click reaction and the lower yields observed with the preparation of the azido acyclic nucleoside, these conjugates were not further pursued. The propargylglycine conjugates **3** and **4** were successfully synthesized using CuAAC to form a triazole linkage between the nucleoside and amino acid. The compounds were fully characterized by ^1^H and ^13^C-NMR and HRMS, and the purity was determined by HPLC. Using COSY and NOESY experiments, the structure of **4** was further confirmed. The successful completion of the syntheses of these analogues allows for future comparative antibacterial studies to be performed. 

## Figures and Tables

**Table 1:** Interaction energies in kcal/mol.

Compound	Vacuum Model		Solvated Model	
	Zn^2+^	Total	Zn^2+^	Total
**1b**	−296	−544	nd	nd
**2b**	−293	−529	nd	nd
**3**	−496	−613	−491	−710
**4**	nd	nd	−470	−602

nd = not determined.

## Supplementary Materials

The following are available online, Figures S1 and S2: ^1^H-^1^H COSY and NOESY spectra, Table S1: Conditions for tosylation reaction.
