# Supplementary material for: Design, Modeling and Synthesis of 1,2,3-Triazole-Linked Nucleoside-Amino Acid Conjugates as Potential Antibacterial Agents
Source: Molecules. 2017 Oct 10;22(10):1682. doi: 10.3390/molecules22101682 (PMC6151744; doi:10.3390/molecules22101682)
Supplement: Supplementary file 1 [file molecules-22-01682-s001.pdf]

Supplementary Material

Design, Modeling and Synthesis of 1,2,3-Triazole-linked Nucleoside-Amino Acid Conjugates as Potential Antibacterial Agents

Sarah N. Malkowski, Carolyn F. Dishuck, Gene G. Lamanilao, Carter P. Embry, Christopher S. Grubb, Omair Arain, Mauricio Cafiero and Larryn W. Peterson *

Department of Chemistry, Rhodes College, 2000 North Parkway, Memphis, TN 38112;
* Correspondence: petersonl@rhodes.edu; Tel.: +01-901-843-3545

Table S1. Reagents and results for the formation of the acyclic tosylate, **6**

Trial	Reagents	Reaction Conditions	Workup and purification	Result
**1**	**5**, TsCl, pyr	0° C to rt, 16 h	Added MeOH, Conc.<br>Column 1: CH_2Cl_2:MeOH<br>Column 2: Hex:EtOAc	12%
**2**	**5**, TsCl, pyr	0° C to rt, 17.25 h	Diluted with CH_2Cl_2<br>Wash: HCl, brine<br>Column: CH_2Cl_2:MeOH	9%
**3**	**5**, Ts_2O, pyr	0° C to 35° C, 4.5 h	Diluted with CH_2Cl_2<br>Wash: HCl, brine, $NaHCO_3$<br>Column: CH_2Cl_2:MeOH	23%
**4**	**5**, Ts_2O, pyr	0° C to rt, 16.5 h	Diluted with CH_2Cl_2<br>Wash: HCl, brine, $NaHCO_3$	0%
**5**	**5**, TsCl, pyr	0° C to rt, 14.75 h	Conc.<br>Column 1: CH_2Cl_2:MeOH<br>Column 2: CH_2Cl_2:MeOH	56%, impure

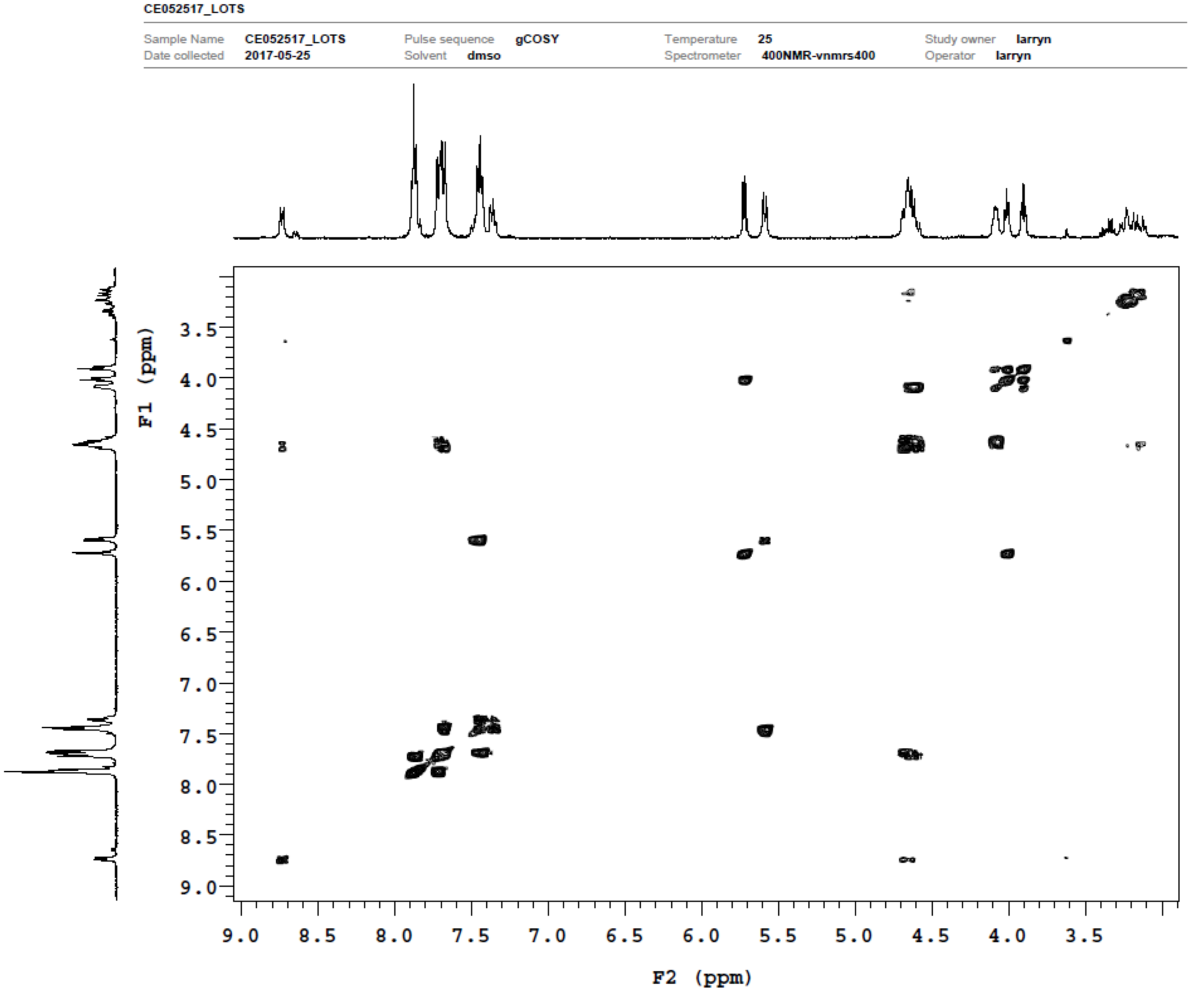

Figure S1. ^1H-^1H COSY of **4**.

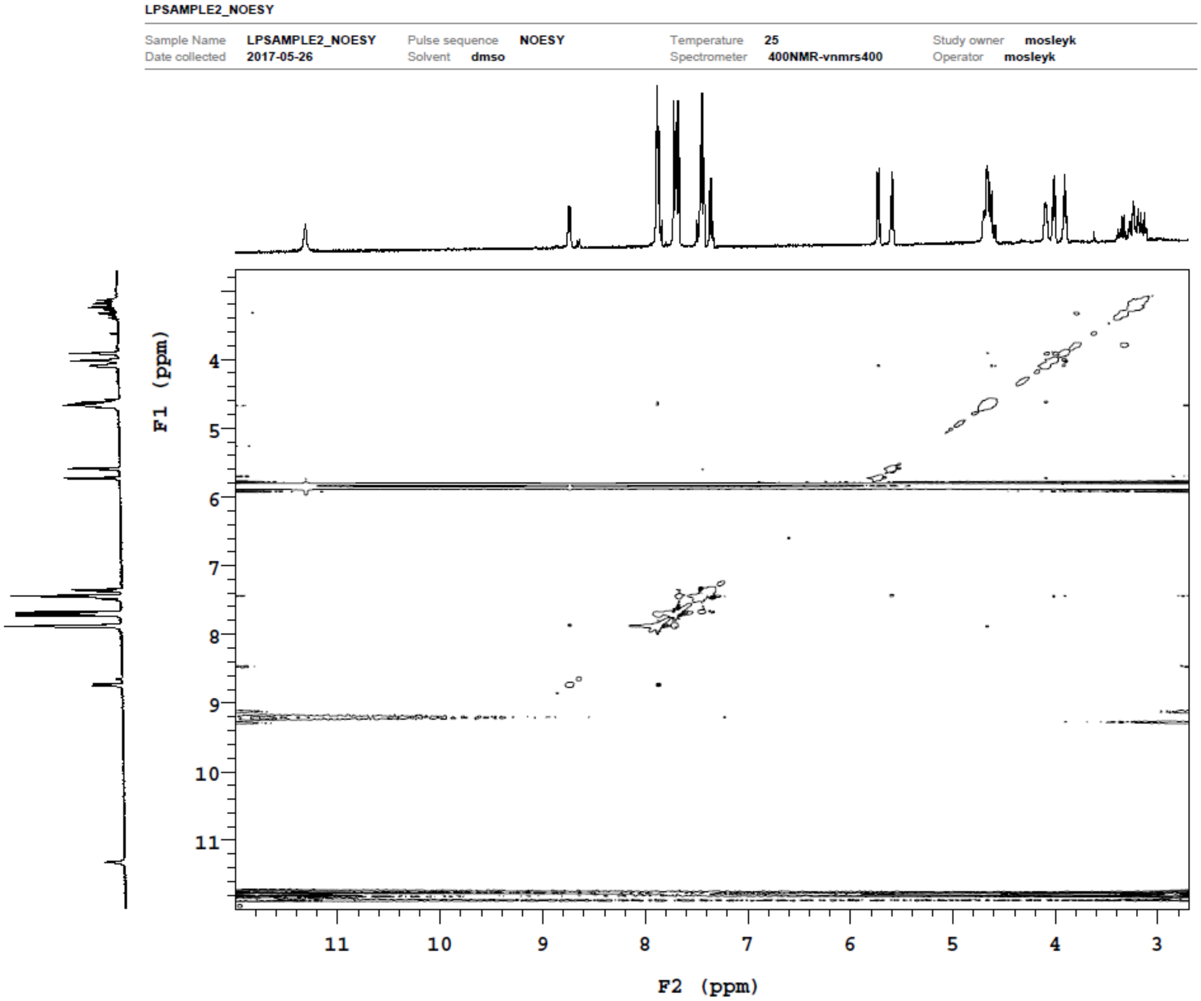

Figure S2. NOESY spectrum of **4**.
